# Social Media Use Time and Mental Health of Young Adults: A Cross‐Sectional Study in Bangladesh

**DOI:** 10.1002/hsr2.71117

**Published:** 2025-07-23

**Authors:** Sudipta Das, Abdullah Al Zubayer, Marzia F. Snigdha, Md. Fahim Uddin, Mubin Khan Afridi, Kazi F. J. Kanak, Mohammad Kibria, Afroja Akter, Israt Jahan, Ashfia M. Rafa, Hasan M. Kamran, Safayet Jamil, Mohammad S. Biswas

**Affiliations:** ^1^ Department of Public Health and Informatics Bangabandhu Sheikh Mujib Medical University Dhaka Bangladesh; ^2^ Department of Sociology University of Barishal Barishal Bangladesh; ^3^ Department of Public Health State University of Bangladesh Dhaka Bangladesh; ^4^ Department of Economics National University Gazipur Bangladesh; ^5^ Graduate School of International Cooperation Studies Kobe University Kobe Japan; ^6^ Department of Anthropology Shahjalal University of Science and Technology Sylhet Bangladesh; ^7^ Department of Sociology Shahjalal University of Science and Technology Sylhet Bangladesh; ^8^ Department of Mass Communication and Journalism Comilla University Cumilla Bangladesh; ^9^ Community Nutrition Department Bangladesh University of Health Sciences Dhaka Bangladesh; ^10^ Department of Law Jagannath University Dhaka Bangladesh; ^11^ Department of Public Health Daffodil International University Dhaka Bangladesh; ^12^ Department of Public and Community Health, Faculty of Medicine and Health Sciences Frontier University Garowe Puntland Somalia; ^13^ Department of Biochemistry and Biotechnology University of Science and Technology Chittagong Bangladesh

**Keywords:** anxiety, Bangladesh, depression, social media, young adults

## Abstract

**Background:**

Social media (SM) use has gained much popularity among young adults, which could impact their mental health. The association between SM use time and young adults' mental health in Bangladesh has been less researched.

**Aim:**

To determine the association between SM use time and mental health among young adults in Bangladesh.

**Methods:**

Cross‐sectional data were collected from 440 young adults aged 18–35 from two districts in Bangladesh: Dhaka and Cumilla. Data were collected using a self‐reported questionnaire, including their socio‐demographic characteristics, SM use time, and mental health disorders—depression and anxiety. Depression and anxiety were assessed by PHQ‐9 and GAD‐7, respectively. SPSS version 22 was used to perform data analysis.

**Results:**

Of the 440 participants, 29.5% used SM for < 2 h/day, 36.6% used for 2–4 h/day, and 33.9% used for > 4 h/day. The crude analysis showed that participants who used SM for > 4 h/day had significantly higher odds of depression than those who used < 2 h/day (OR = 2.094, 95% CI: 1.245, 3.522, *p* = 0.005). The association also remains significant after adjusting for socio‐demographic variables (OR = 2.158, 95% CI: 1.241, 3.753, *p* = 0.006). Also, this study showed that using SM for > 4 h/day was associated with increased odds of anxiety both in crude and adjusted models (OR = 1.864, 95% CI: 1.041, 3.337, *p* = 0.036) and (OR = 1.945, 95% CI: 1.054, 3.587, *p* = 0.033), respectively.

**Conclusion:**

This study shows that using SM for more than 4 h a day is significantly associated with higher odds of depression and anxiety among young adults. A more robust study should be carried out to determine the setting limits for daily SM use that could help reduce the burden of mental health disorders among Bangladeshi young adults.

## 1 Introduction

Social media (SM) refers to online platforms that enable users to interact virtually [1]. There are different types of SM platforms. The popular SM platforms are Facebook, Twitter, YouTube, WhatsApp, TikTok, Instagram, and Snapchat [2]. People use SM platforms for different purposes, such as expressing their thoughts and opinions, communicating with friends and families, entertainment, and professional networking [2, 3]. A previous study reported that more than 90% of college students used SM to communicate with their friends and families [4]. SM platforms are also used for business purposes. Many companies use SM for promoting their brands [5].

Over the past few years, the number of SM users has increased notably. The global SM statistics research 2024 reported that nearly 62% of the world population uses SM, and around 266 million new users were added in 2023 [6]. In recent years, SM has become more popular among young adults, who spend a significant amount of time on different SM platforms daily [2, 7]. In 2016, more than 97% of young adults aged 18–24 years in the United States used at least one SM platform on a regular basis, an increase in users from 89.42% in 2014 to 97.5% in 2016 [8]. Recent statistics showed that the number of SM users in Bangladesh is about 44.7 million, and the majority of the users are males and young adults [9].

Young adults benefit from using SM platforms by connecting with distant friends and family and by being exposed to current events. However, SM use is associated with different adverse mental health outcomes. Studies showed that SM use is associated with mental health disorders, like depression, anxiety, and reduced self‐esteem [10, 11, 12]. Spending too much time on the SM platform disturbs the regular daily routine. Moreover, exposure to misinformation from SM platforms sometimes leads to mental health problems [13]. There is no specific timeframe for how long an individual should spend on SM on a typical day. A longitudinal cohort study with 6595 adolescents aged 12–15 years showed that participants who spend more than 3 h a day using SM are at increased risk for mental health problems, including internalizing and externalizing problems [14]. Another study conducted with 449 young adults in Bangladesh during the second wave of the COVID‐19 pandemic reported that a high level of total media exposure (> 4 h/day) was significantly associated with anxiety [10]. In Bangladesh, where the present study was conducted, the prevalence of mental health problems, like stress, depression, and anxiety, has increased among young adults over the past few years [15, 16]. Previous Bangladeshi studies also highlighted the risk factors of mental health problems among young adults, like Facebook addiction [17], problematic internet use [16], physical inactivity [18], and different socio‐demographic factors [15, 19]. However, there is a relative lack of studies investigating the association between SM use time and young adults' mental health problems in southern Asian countries like Bangladesh. Therefore, the present study aimed to determine the association between SM use time and mental health problems (depression and anxiety) among young adults in Bangladesh.

## 2 Methods

### 2.1 Study Design and Population

A cross‐sectional study was conducted between February and March 2024 among young adults aged 18–35 who resided in two districts in Bangladesh: Dhaka and Cumilla. The inclusion criteria for this study were [1]: being young adults aged 18–35 years [2], using any of the SM platforms [3], being interested in participating voluntarily. We excluded those who were unable to participate due to physical and mental incompetence or unwillingness.

### 2.2 Sample Size and Sampling

The standard formula for observational studies, *n* = [*Z* ^2^*p*(1‐*p*)]/*e* ^2^, was used to calculate sample size. The following assumptions were made for sample size calculation: prevalence of SM use among young adults (*p*) as 50%, the critical value (*Z*) at 95% confidence interval as 1.96, and the margin of error (*e*) as 5%. Based on these assumptions, the minimum required sample for this study was to be 383.

Due to a lack of a sampling frame of the young adult population, participants were recruited using the convenience sampling technique. Due to the constraints of time, resources, and the descriptive exploratory nature of the study, a convenience sampling strategy was used. To effectively limit potential selection bias, we implemented strict inclusion criteria and ensured that participant demographics approximately reflected both rural and urban populations of Bangladesh. Participants were invited to respond and participate in this study by verbal communication at local gathering places such as local markets, parks, and playgrounds. Overall, 500 participants were approached to participate in the study, of which 440 participants responded and were included in the analysis.

### 2.3 Data Collection

A face‐to‐face interview was conducted to collect data from the participants. Data were collected using a predesigned semistructured questionnaire, which consisted of four parts [1]: socio‐demographic characteristics [2], SM use time [3], patient health questionnaire (PHQ‐9) to assess depression, and [4] generalized anxiety disorder (GAD‐7) to assess anxiety.

#### 2.3.1 Socio‐Demographic Characteristics

Socio‐demographic characteristics included age, gender, occupation, monthly family income, education level, marital status, residence, and smoking habit.

#### 2.3.2 SM Use Time

SM use time was assessed by asking, “During the last 7 days, how much time did you spend on SM during a typical day?”. Responses were recorded in minutes, later converted to hours, and categorized as < 2 h/day, 2–4 h/day, and > 4 h/day.

#### 2.3.3 Depression

The PHQ‐9 is a popular tool to assess depression among different populations [20]. This scale was also validated for the Bangladeshi population [21]. For this study, we used the Bengali version of the PHQ‐9 to assess depression in the participants. It is a nine‐item self‐reported scale, and responses were recorded on a four‐point Likert scale (0 = not at all, 1 = several days, 2 = more than half of the days, and 3 = nearly every day), and the scores range from 0 to 27. A score of ≥ 10 was considered to be having depression. We found good reliability of the scale in this study (Cronbach's *α* = 0.860).

#### 2.3.4 Anxiety

The GAD‐7 is a seven‐item self‐reported scale for assessing anxiety [22]. In this study, we used the Bengali version of the GAD‐7 [23] to assess anxiety of the participants. Responses were recorded on a four‐point Likert scale (0 = not at all, 1 = several days, 2 = more than half of the days, and 3 = nearly every day), and the scores range from 0 to 21. A score of ≥ 10 was considered the cut‐off score for experiencing anxiety. The present study found an excellent reliability of the scale (Cronbach's *α* = 0.901).

### 2.4 Data Analysis

Data analysis was performed using SPSS (version 22.0, IBM). We performed descriptive statistics, such as mean and standard deviation for continuous variables like age and frequency, and percentage for categorical variables. The outcome variables of this study, depression and anxiety, were binary. Therefore, binary logistic regression was employed to determine the relationship between SM use time and depression, and between SM use time and anxiety. Before fitting logistic regression models, assumptions were checked and found to be satisfactory. At first, we employed two crude models independently for the outcome variables, depression and anxiety, with SM use time as the predictor. Then, we employed two adjusted models: one for the outcome variable, depression, and another for the outcome variable, anxiety. In the adjusted model, variables such as age, gender, occupation, income, education, marital status, residence, and smoking habit were included, as these variables might influence the association between SM use time and mental health problems. We reported the regression analysis results by odds ratio with a 95% confidence interval. Odds ratio was used to compare the strength of association between the outcome variable and the predictor. To determine a statistically significant association, we considered the confidence interval and associated *p*‐value of the test.

### 2.5 Ethical Approval

This study was conducted following the Helsinki Declaration and the ethical guidelines for studies involving human participants. Ethical approval was obtained from the institutional review board of the Khwaja Yunus Ali University, Sirajganj‐6751, Bangladesh. Before collecting data, informed written consent was obtained from each of the participants. Participants were well briefed about the study objectives, procedures, and information confidentiality. Participants were also assured that they could withdraw from the study at any moment without any justification.

## 3 Results

Table 1 demonstrates the socio‐demographic characteristics of the participants. Among the 440 participants included in this study, the majority (63%) were in the 18–25 age group, about 29% in the 26–30 age group, and the rest (8%) in the 31–35 age group. Around 54% of the participants were males and 46% were females. Most participants (59%) resided in urban areas, and the rest (41%) were from rural areas. These numbers closely reflect the population statistics of Bangladesh, which indicate an urban‐to‐rural ratio of approximately 68%–32%, according to the Population and Housing Census 2022 [24]. Around 56% were students, while approximately 32% were employed. Most participants (40%) had a family income of less than 25,000 Bangladeshi Taka (BDT), followed by around 36% having a family income of 25,000–50,000 BDT. Most of the participants (83%) in this study were nonsmokers. The participants were divided into three categories based on their duration of SM use. Most participants (36.6%) used SM for 2–4 h/day, while 33.9% used SM for > 4 h/day, and 29.5% used SM for < 2 h/day.

**Table 1:** Descriptive statistics of study participants (*N* = 440).

Variables	Category	Frequency	Percentage
Age (in years)	18–25	278	63.2
	26–30	126	28.6
	31–35	36	8.2
Gender	Male	237	53.9
	Female	203	46.1
Occupation	Students	246	55.9
	Unemployed	40	9.1
	Employed	142	32.3
	Housewife	12	2.7
Monthly family income (BDT)	Less than 25,000	176	40.0
	25,000–50,000	160	36.4
	More than 50,000	104	23.6
Education level	Secondary or below	51	11.6
	Higher secondary	172	39.1
	Graduate or above	217	49.3
Marital status	Unmarried	339	77.0
	Married	101	23.0
Residence	Urban	261	59.3
	Rural	179	40.7
Smoking habit	Yes	75	17.0
	No	365	83.0
SM use time	< 2 h/day	130	29.5
	2–4 h/day	161	36.6
	> 4 h/day	149	33.9

**Figure 1:**
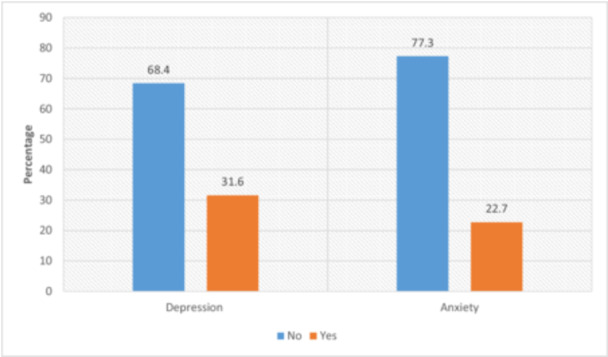
Depression and anxiety prevalence among study participants.

As per the PHQ‐9 score, 68.4% of the participants had no depression (PHQ‐9 score of < 10), while 31.6% were found to have depression (PHQ‐9 score of ≥ 10). Based on the GAD‐7 score, 77.3% of the participants had no anxiety (GAD‐7 score ≥ 10), while 22.7% had anxiety (GAD‐7 score ≥ 10). The prevalence of depression and anxiety among participants is displayed in Figure 1.

Table 2 displays the binary logistic regression models to predict the association between the participants' depression and SM use time. In model 1 (crude analysis), we found that participants who reported using SM for > 4 h/day had higher odds of depression compared to those who used SM for < 2 h/day (odds ratio [OR] = 2.094, 95% confidence interval [CI]: 1.245, 3.522, *p* = 0.005). The model 2 which was adjusted for age, gender, occupation, family income, education, marital status, residence, and smoking habit, showed that participants who reported using SM for > 4 h/day had higher odds of depression compared to those who used SM for < 2 h/day (OR = 2.158, 95% CI: 1.241, 3.753, *p* = 0.006). However, we found no significant association between SM use time and depression in both models for SM use time 2–4 h/day.

**Table 2:** Association between social media use time and depression.

	Social media use time	OR	95% Cl		
			Lower	Upper	*p*‐value
Model 1	< 2 h/day	Ref.			
	2–4 h/day	1.397	0.827	2.361	0.212
	> 4 h/day	2.094	1.245	3.522	0.005
Model 2	< 2 h/day	Ref.			
	2–4 h/day	1.444	0.830	2.512	0.193
	> 4 h/day	2.158	1.241	3.753	0.006

*Note:* Model 1: crude analysis (dependent variable: depression). Model 2: adjusted for variables: age, gender, occupation, income, education, marital status, residence, smoking habit (dependent variable: depression). Abbreviations: CI, confidence interval; OR, odds ratio.

Table 3 shows the binary logistic regression models to predict the association between the participants' anxiety and SM use time. Model 1 (crude analysis) showed that the odds of anxiety were significantly higher among participants who reported using SM for > 4 h/day compared to those who used SM for < 2 h/day (OR = 1.864, 95% CI: 1.041, 3.337, *p* = 0.036). The adjusted model 2 also showed that participants who reported using SM for > 4 h/day had higher odds of anxiety than those who used SM for < 2 h/day (OR = 1.945, 95% CI: 1.054, 3.587, *p* = 0.033). Comparing people who used SM for 2–4 h/day to those who used it for < 2 h/day, no significant association was found between anxiety and SM use for < 2 h/day in both models.

**Table 3:** Association between social media use time and anxiety.

	Social media use time	OR	95% Cl		
			Lower	Upper	*p*‐value
Model 1	< 2 h/day	Ref.			
	2–4 h/day	1.465	0.814	2.636	0.203
	> 4 h/day	1.864	1.041	3.337	0.036
Model 2	< 2 h/day	Ref.			
	2–4 h/day	1.500	0.813	2.768	0.194
	> 4 h/day	1.945	1.054	3.587	0.033

*Note:* Model 1: crude analysis (dependent variable: anxiety). Model 2: adjusted for variables: age, gender, occupation, income, education, marital status, residence, smoking habit (dependent variable: anxiety). Abbreviations: CI, confidence interval; OR, odds ratio.

## 4 Discussion

The present study investigated the association between SM use time and mental health problems among young adults in Bangladesh. The findings of this study showed that more than one‐third of the participants (33.9%) used SM for more than 4 h on a typical day, while nearly 37% of the participants used SM for 2–4 h a day, and approximately 30% used it for less than 2 h a day. A previous study conducted with 449 Bangladeshi young adults during the second wave of the COVID‐19 pandemic showed that 44.5% of the participants had low exposure (< 2 h/day) to different media, while 39.2% had medium (2–4 h/day) and 16.3% had high exposure (> 4 h/day), respectively [10]. Another study in Bangladesh involving 385 university students reported that nearly 55% of the participants used Facebook, an SM platform, for more than 2 h a day [25]. These findings underscore the significant amount of time young adults spend on SM platforms, with usage patterns varying across studies. While some use SM for longer periods, others spend comparatively less time on these platforms. This diversity in SM use emphasizes the need to explore the potential impacts of SM use on mental health issues, considering different levels of SM exposure among young adults in Bangladesh.

The findings of this study further showed that about one‐third of the participants (31.6%) had depression, and almost one‐fourth of the participants (22.7%) had anxiety. In the past few years, we have seen an increase in the prevalence of different mental health problems in Bangladesh, particularly among young adults. A previous study with 590 Bangladeshi university students (mean age: 24.12 years), young adults in age, reported that the prevalence of depression and anxiety was 52.2% and 58.1%, respectively [26]. Rahman et al. conducted a study with 365 university students in Bangladesh during the COVID‐19 pandemic and reported that the prevalence of depression and anxiety was 30.41% and 43.29%, respectively [27]. Another Bangladeshi study with 791 participants aged 15–40 years by Islam et al. reported that the prevalence of depression and anxiety was 38% and 63%, respectively [28]. The conflicting findings could be due to either different age populations or different study periods, or assessing depression and anxiety by other scales.

This study showed that SM use time was significantly associated with depression and anxiety among young adults. Specifically, participants who used SM for more than 4 h a day were more likely to experience depression and anxiety compared to those who used less than 2 h a day. This finding was somewhat consistent with the findings of Riehm et al. [14] and Pitol et al. [10], which reported that SM use time was associated with depression and anxiety. There is ample evidence in the literature that SM use has adverse effects on mental health. Young adults use SM as a substitute for offline interaction. The increased amount of time spent on SM hampers mental well‐being in many ways, as one can get exposed to misinformation through SM platforms and may feel pressure to check updates on SM platforms frequently and form opinions on SM platforms regularly. However, there is no set limit for SM use. SM use could be problematic if it affects an individual's daily life. In the literature, we find guidelines about leisure time screen activities. Many experts suggest that recreational screen‐time should be within 2–4 h a day for adults aged over 18 years [29].

SM use could benefit young adults in several ways, such as keeping them up‐to‐date about recent events, job searching, and entertainment [2, 3]. Extended use of SM has been shown to negatively affect the mental health of young adults. Previous studies also reported that SM use is associated with other mental health issues, such as loneliness and social isolation [30, 31]. These negative concerns associated with extended use of SM and the absence of established guidelines for SM use time highlight the importance of more research to determine optimal usage limits for SM. Determining time limits for SM use could eventually better safeguard the mental well‐being of the users and foster healthier online habits in today's digital age.

This study had several limitations, which should be considered when interpreting its findings. This study utilized self‐reported data, which could introduce biases such as memory recall bias and social desirability bias, and impact the findings. Self‐reported measures of SM usage are prone to recall bias, as participants may not accurately remember or might misestimate the time they spend on SM platforms. This could result in either an overestimation or an underestimation of actual usage, potentially influencing the observed associations with their mental health outcomes. Further, as this study was cross‐sectional, it could not infer a causal association between SM use time and mental health problems. However, this study has several implications for future research and public health interventions. Randomized control trials on this topic seem to be unethical. Other study designs, such as longitudinal studies, could be further conducted to determine the causal association between SM use time and mental health problems. To better understand the potential causal pathways, future research should utilize longitudinal designs that will help clarify whether depression and anxiety precede or result from SM use. However, this study could act as a baseline study for future studies. For public health interventions, this study highlights the importance of considering SM use time while designing mental health interventions for young adults in Bangladesh.

## 5 Conclusion

This study found that Bangladeshi young adults spend a considerable amount of time on various SM platforms, with daily SM use of more than 4 h being significantly associated with higher odds of depression and anxiety. Given these findings, this study highlights the importance of optimal SM use by young adults to reduce the risk of mental health issues and improve overall well‐being in this population. Further, there is a critical need for more comprehensive research to establish guidelines for daily SM usage that could help mitigate the mental health challenges faced by young adults in Bangladesh, as well as other settings with a similar background.

## Author Contributions

**Sudipta Das:** formal analysis, writing – original draft, writing – review and editing, conceptualization, methodology, data curation. **Abdullah Al Zubayer:** writing – review and editing, project administration, writing – original draft, conceptualization, methodology. **Marzia F. Snigdha:** writing – original draft, conceptualization, validation, data curation, writing – review and editing. **Md Fahim Uddin:** writing – original draft, writing – review and editing, conceptualization, validation, data curation. **Mubin Khan Afridi:** writing – original draft, visualization, writing – review and editing. **Kazi F. J. Kanak:** writing – original draft, methodology, writing – review and editing. **Mohammad Kibria:** writing – original draft, writing – review and editing. **Afroja Akter:** writing – review and editing. **Israt Jahan:** writing – original draft, writing – review and editing. **Ashfia M. Rafa:** writing – review and editing. **Hasan M. Kamran:** writing – review and editing. **Safayet Jamil:** writing – review and editing. **Mohammad S. Biswas:** investigation, supervision, writing – review and editing.

## Conflicts of Interest

The authors declare no conflicts of interest.

## Transparency Statement

The lead author Abdullah Al Zubayer affirms that this manuscript is an honest, accurate, and transparent account of the study being reported; that no important aspects of the study have been omitted; and that any discrepancies from the study as planned (and, if relevant, registered) have been explained.

## Data Availability

The data that support the findings of this study are available from the corresponding author upon reasonable request.
